# Effect of Aging on the Lower Limb Kinematics in Karate Practitioners: Comparing Athletes and Their Senseis

**DOI:** 10.1155/2019/2672185

**Published:** 2019-06-04

**Authors:** Marco A. C. Branco, António M. V. VencesBrito, Mário A. Rodrigues-Ferreira, Gonçalo A. C. Branco, Ewa Polak, Wojciech J. Cynarski, Wąsik Jacek

**Affiliations:** ^1^Sport Sciences School of Rio Maior, Polytechnic Institute of Santarém, Rio Maior, Portugal; ^2^Biomechanics and Functional Morphology Laboratory, Interdisciplinary Centre for the Study of Human Performance (CIPER), Faculty of Human Kinetics, University of Lisbon, Lisbon, Portugal; ^3^International Martial Arts and Combat Sports Scientific Society (IMACSSS), Rzeszow, Poland; ^4^IPSantarem Research Unit (UI-IPS) - Physiology of Exercise and Sport Sciences, Applied Psychology, Santarém, Portugal; ^5^Life Quality Research Centre (CIEQV), Santarém, Portugal; ^6^Physiotherapy and Sports Centre, Rzeszow University of Technology, Rzeszow, Poland; ^7^Faculty of Physical Education, University of Rzeszow, Rzeszow, Poland; ^8^Institute of Physical Education, Tourism and Physiotherapy, Jan Długosz University in Czestochowa, Czestochowa, Poland

## Abstract

With the life expectancy increasing, older adult population has gained the attention of many researchers. Aging is known to lead to a general decline in bodily functions, which affect the quality of life. The aim of this study was to analyze how the aging process affects veteran active karate practitioners, in the kinematic and temporal structure of the frontal kick. Nine black belt karate practitioners over 50 years old and 24 black belt karate practitioners, aged between 20 and 30 years old, all male, performed the frontal kick *mae-geri*. Results showed that knee is the structure that holds most differences between young and veterans, both for linear and for angular variables during the *mae-geri* performance. Statistical differences were found in linear velocity for the knee; linear acceleration of the knee, hip, and RASIS; maximum angular velocity for knee and hip; maximum angular acceleration for ankle and hip; and in the range of motion of knee. The temporal variables show differences, between groups, in maximum linear velocity, maximum linear acceleration, and maximum angular acceleration. However, no differences were found between groups for the time before contact in the maximum linear and angular acceleration, which allow us to remark both the effects of the aging process and the effect of training. This study corroborates the ability of older people to achieve benefits from sports practice, achieving higher efficiency than the younger adults in task execution, but using different motor control strategies.

## 1. Introduction

In actual days, with the life expectancy increasing, older adult population has gained the attention of many researchers. Aging is known to lead to a general decline in bodily functions, which affect the quality of life. Muscle function begins to slow the contraction from 40 to 50 years and decreases muscle strength 1 to 1.5% per year after 60 years [1–3]. Older adults do less work for lower and fast angular velocities [4] and use less power for slow, moderate, and fast angular velocities [5], than young adults. These losses occur as age increases. The sedentary lifestyle is one factor that exacerbates these functional losses; however, the physical activity and exercise appear as an inversion factor and not only reverse this decline but also promote an increase in functional capacity at the neuromuscular level. Although athletic performance is diminished in older adults, karate coaches or senseis are the technical execution model and they need a long time and training to achieve their status. This leads us to a question: do karate young adults have better performance than their older senseis? The aim of this study was to analyze how the aging process affects veteran active karate practitioners, in the kinematic and temporal structure of the frontal kick. Thus, the first hypothesis is that kinematic variables present a worse performance in veteran adults than in young adults. The second hypothesis is that the temporal structure of the frontal kick represents a worse performance in veteran adults than in young adults.

## 2. Methods

### 2.1. Experimental Approach to the Problem

The kinematic methodologies in biomechanics allow collection of variables based on time and position, as is the case of velocity and acceleration, both for linear and angular motion. These variables can be considered as kinematic factors for the evaluation of sports performance, since they accurately quantify the way each subject performs the movement. In this way, it is possible to rigorously compare the execution of specialized motor tasks between subjects. The use of two groups of experienced practitioners allows verification of the changes that the years of practice and the age have in these variables.

### 2.2. Subjects

Black belt karate practitioners (*N* = 33) with more than 10 years of practice volunteered to participate in the study. Two groups were formed to accomplish the study objectives: one with nine veteran karate practitioners with 54.2 ± 3.9 years old (VetK) and the other group with twenty-four karate practitioners with 23 ± 5.8 years old (YgK).

All participants signed an informed consent document and were in perfect health and without history of locomotors system disease. The participants' characteristics are shown in Table 1. This study was approved by the Scientific Committee of the Sport Sciences School of Rio Maior, Polytechnic Institute of Santarém, Portugal, and was conducted according to the Helsinki Declaration.

### 2.3. Procedures

**Figure 1:**
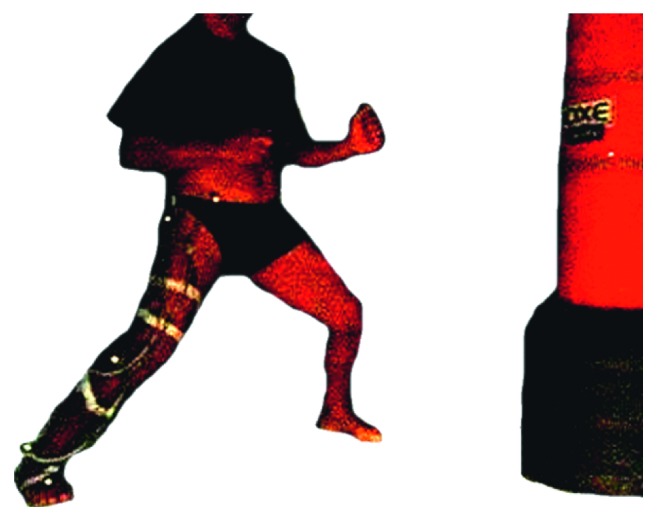
Illustration of the starting position, with the typical karate static stance of *zenkutsu-dachi*, and the target bag.

Each participant did a single measurement session where the collection of kinematic data was performed. The task consisted in the execution of a frontal kick, known as *mae-geri* kick, into a vertical training bag. Participants performed the technique to an area placed at a height of 90 cm from the ground, and they were distanced from the bag at a lower limb length. The starting position were the typical karate static stance of *zenkutsu-dachi* (Figure 1), and they kicked in response to a sound stimulus, which was synchronized with a trigger connected to the video recorder device. Each participant performed the *mae-geri* kick three times, the fastest and strongest as possible and made the impact with the *koshi* (metatarsals), on the bag. Between repetitions, there was a rest period of 30 seconds.

To build the biomechanical model, five reflective markers were placed on the right anterior superior iliac spine (RASIS), the prominence of the greater trochanter external surface (hip), the lateral epicondyle of the femur (knee), the distal apex of the lateral malleolus (ankle), and the dorsal aspect of the second metatarsal head (forefoot) of the right lower limb [6]. A high-speed camera (Casio EX-FH20), positioned perpendicular to the plane of motion, collected images at a sampling frequency of 210 Hz. The images were initially cut and analyzed with the software Ariel Performance Analysis System (APAS, Ariel Dynamics-2003). The remaining calculations were automatically processed in the MATLAB software (The MathWorks, Inc., Natick, Massachusetts, USA).

**Figure 2:**
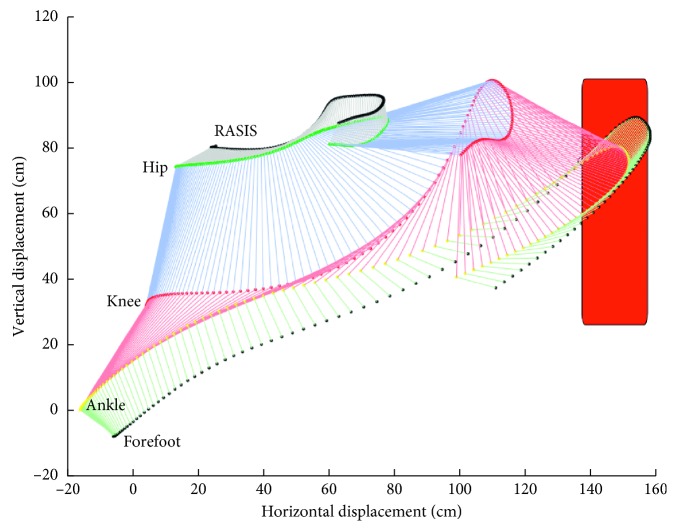
Illustration of the virtual segments of the right pelvis, thigh, leg, and foot during the task execution. First left lines represent the starting position of the right lower limb.

The virtual lab was calibrated using eight control points, with specific coordinates (*x*, *y*), through Digitize module of the APAS system. Once the markers were identified and digitalized in all the frames, the reconstruction of the trajectory was made with the Transform module of the APAS software, through the direct linear transformation-DLT algorithm [7]. These trajectories were smoothed, using a low-pass digital filter with a 5 Hz cutoff [8] in the Filter module of the APAS software. The reconstruction of the virtual segments considering point coordinates was done in MATLAB software (Figure 2).

The variables under study are divided in linear, angular, and temporal parameters. The linear parameters include the maximum linear velocity and the maximum linear acceleration corresponding, respectively, to its maximum peak occurred during the task execution, for all collected points. The angular parameters include the range of motion of the hip, knee, and ankle joints, which was calculated by subtracting the minimum to the maximum angle obtained during task; the joints angle in the initial stance and in the moment of contact with the bag; and also were collected the maximum peak of angular velocity and angular acceleration for the hip, knee, and ankle, obtained during the task. The temporal parameters were divided into variables where the time begins to count from the moment of the stimulus and those in which the time begins to count back from the moment of contact with the bag. Thus, considering the first group of temporal variables, were collected the time in which the movement began after the stimulus, as well as the time in which the maximum peaks of linear velocity and acceleration for all anatomical points, and the peaks of angular velocity and acceleration for all joints, occurred. On the other hand, for the second group of temporal variables, was measured the time from the referred maximum peaks until the moment of contact with the bag.

### 2.4. Statistical Analysis

Descriptive data are presented as means and standard deviations. Normality of the data distribution was tested and not assumed for all variables. *U*-Mann–Whitney was performed to examine the differences between the two groups on the kinematic variables. The level of statistical significance was set to *α* ≤ 0.05. Statistical analysis was performed with Statistical Package for the Social Sciences (IBM SPSS Statistics 25.0 for Windows®, Chicago, USA).

## 3. Results

Significant differences were found between veterans and young practitioners group for black belt level, years of practice, age, and body fat, with VetK having higher values (Table 1).

### 3.1. Segmental Movement Results

Table 2 shows the collected data of all kinematic variables as well as the significant differences between the VetK and the YgK.

The kinematic variables show the knee as the structure that holds most differences between young and veterans, both for linear and for angular variables. For linear kinematics, the young practitioners achieve higher values in maximum linear velocity only for the knee marker, differing 0.30 m/s for the veteran practitioners' group (*p* ≤ 0.05). Differences between groups in the linear acceleration were found for knee, hip, and RASIS markers, differing, respectively, 11.56 m/s^2^, 4.93 m/s^2^, and 3.47 m/s^2^ (*p* ≤ 0.001).

The initial angle of the knee differs between groups with a higher angle for young practitioners (*p* ≤ 0.001), having a higher knee extension than the veterans' group by a mean value of five degrees. In the contact angle, joints show no differences between groups; however, minimum and maximum angular peaks and the range of motion of the knee joint had differences between groups, with the young practitioners making greater magnitudes of knee flexion (*p* ≤ 0.01) and knee extension (*p* ≤ 0.01), and therefore, a greater range of motion (*p* ≤ 0.001) for this joint.

The comparisons of the maximum angular velocity show differences between groups only in knee and hip joints (respectively, *p* ≤ 0.05 and *p* ≤ 0.03), where the younger group performed more, 49 deg/s and 143.5 deg/s, respectively, for knee and hip joints than the veterans' group. In the maximum angular acceleration, the veterans group performed significant lower magnitudes for ankle and hip joints (*p* ≤ 0.05) than the younger group.

In Table 3 are presented the temporal events of kinematic variables.

No differences were found in initial time; however, the maximum linear velocity time is significantly different between groups for the forefoot, ankle, and knee markers (*p* ≤ 0.02), where young group practitioners reach maximum velocity about 50 ms earlier than the veterans' group, in the three anatomical points. For the maximum linear acceleration time, the young group also reaches the peak about 50 ms before veterans' group for forefoot and ankle, 65 ms earlier for knee, and about 90 ms earlier for hip and RASIS markers.

In the maximum angular velocity time, the young group reaches the peak 67 ms before the veteran group for the hip joint and 43 ms for the knee joint. For the maximum angular acceleration time of the hip and knee joints, the veteran group achieves it 49 ms and 32 ms after the young group, respectively.

Concerning the time to contact of the maximum angular velocity, the peak of the ankle happened 32 ms away from the contact in the veterans' group than in the young group. Another significant difference was found at time to contact of the maximum linear velocity peak of the ankle; however; the mean time is similar between groups, showing that these statistical differences are essentially due to the dispersion of the data. It should be noted that this dispersion of the data is always greater in the group of young practitioners than in the group of veterans.

## 4. Discussion

The purpose of this study was to analyze how the aging process affects veteran active karate practitioners, in the kinematic and temporal structure of the frontal kick. Aging is a biological process characterized by physiological, mechanical, and other adaptations, which result from the passage of time. In this study, the veterans' group shows a higher amount of fat mass which is in consonance with Tian et al. [9] who claim that body fat remains stable until 40 years old and increases thereafter. Nevertheless, considering the age, both groups show an assessment of “Good” according to the fat mass tables assessment [10], demonstrating that both groups are at the same level of body fat mass. This information may mean that fat mass differences between groups may not be a factor that influences the motor performance assessed in this study; however, the authors suggest that this issue should be considered in future studies. Other significant differences were found in the characterization variables (Table 1), which are in the background of this study, namely, the years of practice and the black belt level. These two variables are related, because in most karate black belt graduation syllabus, there are criteria for the time in the last graduation, and a minimum age to have access to higher levels of graduation. This means that only older people will have higher black belt graduations. So, these differences were expected and consolidate the groups under analysis, which underlies the interest in keeping the research in this topic.

Throughout life, the neuromuscular and musculoskeletal systems of humans undergo different biological processes, which lead to growth, development, maturation, a peak of performance, and later in the decline of their functions, especially in sedentary individuals [11, 12]. After the age of 40, the decline of neuromuscular functions undergoes a decrease in the velocity of contraction, and Keller and Engelhardt [13] report a reduction in the capacity of force production starting at the age of veterans group, but other studies point to the reduction of this capacity only after 60 years old [3, 12, 14]. Although there is a generalized decline in bodily functions in older people, exercise and sports appear as an inverse feature to this decline [15, 16]. In addition to preventing the deterioration of body functions in the elderly, physical activity is associated with a better quality of life, specifically in physical activity indicators such as leg extension strength or walking speed [17], and individuals with higher levels of physical activity have showed the lowest relation to all-cause mortality [18].

The results of our study have outcomes that allow us to see both the aging process and the effect of training. The aging process can be seen in the magnitude of the kinematic variables, as is the case of the veterans that perform less flexion and extension of the knee and, therefore, less range of motion of this joint. Similar results were found by Begg and Sparrow [19], for walking gait; however, these authors specify that this happens in order to allow a better acceptance of the body weight during that motor task, which happens to have a better damping during contact with the ground. In our task, a lower knee ROM for the veterans' group could be a strategy to increase muscle tone, in order to prepare the muscles for a fast contraction which lead to a fastest kick. However, this statement cannot be confirmed with our kinematic data, leaving this premise open for future studies. Dalton et al. [5] found that older men were 42% less powerful and slower in knee velocity for fast-unconstrained velocity tasks, than young men. In this study, angular velocity of the hip and knee also achieves lower peaks for the veterans' group, with special focus on the hip joint, in which the peak is almost 50% lower than in the youth group. For angular accelerations, the veterans' group performs lower peaks than the young's group, for hip and ankle joints. In these variables, the hip joint is the one with proportionally larger differences between groups.

Considering the linear parameters, maximum linear acceleration peak of the RASIS, hip and knee was significantly lower for veterans, and although in the literature we have not found the effect that aging has on this variable, there is a curiosity, since the veterans significantly accelerate less the proximal body segments, but in the distal ones, the maximum linear acceleration are similar to youth group, which is where contact happens.

The effect of training can be seen in the temporal structure of *mae-geri* kinematics, where veterans group shows a higher efficiency of task execution. Two reasons are pointed to prove this statement: first, although in the veteran's group, the peaks happen later considering the beginning of the movement and most peaks occur simultaneously between groups if we consider the time to contact, and second, because the linear velocity and acceleration in the instant of contact were similar between groups. Specifically, the second reason shows the high efficiency that veterans have in the execution of *mae-geri*, because they do not need to spend as much energy to reach the same magnitude of momentum of the foot in the moment of contact.

## 5. Conclusions

The literature reports that aging leads to an overall decline in body functions; however, exercise and sports practice appears as an inverse feature to this decline. The results of our study have outcomes that allow us to see the aging process and the effect of training. The aging process can be seen in kinematic variables, where linear and angular velocities and accelerations have lower magnitudes for veterans' group when compared with younger group. The effect of training can be seen in the temporal structure of *mae-geri* kinematics, where veterans group shows a higher efficiency of task execution, specifically because those peaks occur simultaneously between groups if we consider the contact time. Nevertheless, we verified the need to conduct future studies on the kinetics of motor tasks in populations with a large number of years of sports practice, in order to verify the effect of aging and the effect of training on those populations.

## Figures and Tables

**Table 1:** Participants data of veterans (VetK) and young practitioners (YgK).

Group	Age (years)^a^	Height (cm)	Weight (kg)	Leg length (cm)	Body fat (%)^a^	Weekly training hours	Years of practice^a^	Black belt level (Dan)^a^
YgK	23.7 ± 5.8	174.2 ± 7.9	73.1 ± 14.6	89.9 ± 5.5	12.9 ± 6.5	7.4 ± 4.4	14.0 ± 4.6	1
VetK	54.2 ± 3.9	173.0 ± 6.6	78.5 ± 7.5	88.4 ± 3.9	20.8 ± 3.0	4.9 ± 1.7	32.8 ± 10.0	5

Note: VetK = veteran karate practitioners; YgK = young karate practitioners. Values are means ± standard deviation. ^a^Statistically significant differences between groups (*α* ≤ 0.05).

**Table 2:** Mean and standard deviation of the kinematic variables in VetK and YgK participants.

		RASIS	Hip	Knee	Ankle	Forefoot
Max. linear velocity (m/s)	YgK	1.90 ± 0.43	2.28 ± 0.57	5.85 ± 1.07^a^	8.46 ± 1.45	9.08 ± 1.67
	VetK	1.74 ± 0.36	2.08 ± 0.40	**5.54** **±** **0.68**	8.45 ± 0.96	8.83 ± 1.11

Max. linear acceleration (m/s^2^)	YgK	**19.24** **±** **5.34**^**a**^	**20.04** **±** **6.32**^**a**^	**71.11** **±** **15.29**^**a**^	95.94 ± 17.30	112.30 ± 23.94
	VetK	**15.77** **±** **3.70**	**15.10** **±** **3.39**	**59.55** **±** **9.66**	98.19 ± 14.82	111.23 ± 15.03

Initial angle (deg)	YgK		131.89 ± 10.40	**170.00** **±** **6.50**^**a**^	109.61 ± 23.32	
	VetK		131.50 ± 4.54	**164.38** **±** **7.79**	108.28 ± 22.22	

Contact angle (deg)	YgK		80.88 ± 26.34	130.52 ± 10.88	101.48 ± 10.98	
	VetK		76.46 ± 27.16	128.91 ± 7.11	96.72 ± 6.89	

Max. peak angle (deg)	YgK		135.04 ± 9.35	**175.40** **±** **6.16**^**a**^	123.44 ± 17.29	
	VetK		133.11 ± 4.23	**171.13** **±** **6.05**	121.64 ± 18.99	

Min. peak angle (deg)	YgK		72.57 ± 24.61	**69.76** **±** **9.79**^**a**^	86.48 ± 7.50	
	VetK		70.57 ± 20.83	**75.31** **±** **6.56**	87.87 ± 5.25	

Range of motion (deg)	YgK		62.47 ± 22.87	**105.64** **±** **11.73**^**a**^	36.96 ± 17.81	
	VetK		62.54 ± 18.88	**95.82** **±** **5.50**	33.77 ± 17.28	

Max. angular velocity (deg/s)	YgK		**340.28** **±** **295.68**^**a**^	**849.30** **±** **174.30**^**a**^	277.67 ± 162.41	
	VetK		**196.80** **±** **161.20**	**800.28** **±** **60.81**	231.23 ± 122.48	

Max. angular acceleration (deg/s^2^)	YgK		**13270.99** **±** **7566.46**^**a**^	10842.34 ± 3607.07	**7736.15** **±** **2735.61**^**a**^	
	VetK		**9620.57** **±** **4484.34**	9903.80 ± 1739.83	**7210.76** **±** **5717.75**	

Note: VetK = veteran karate practitioners; YgK = young karate practitioners. Values are mean ± SD. ^**a**^Statistically significant differences between groups (*α* ≤ 0.05).

**Table 3:** Mean and standard deviation of the temporal events of kinematic variables for VetK and YgK participants.

		RASIS	Hip	Knee	Ankle	Forefoot
Initial time (ms)	YgK	196 ± 59	219 ± 65	314 ± 77	365 ± 72	412 ± 62
	VetK	195 ± 62	224 ± 78	324 ± 67	384 ± 62	435 ± 56

Max. linear velocity time (ms)	YgK	510 ± 99	533 ± 85	**533** **±** **71**^**a**^	**638** **±** **77**^**a**^	**617** **±** **77**^**a**^
	VetK	539 ± 112	568 ± 89	**578** **±** **61**	**686** **±** **64**	**668** **±** **62**

Max. linear acceleration time (ms)	YgK	**503** **±** **137**^**a**^	**556** **±** **151**^**a**^	**647** **±** **99**^**a**^	**695** **±** **78**^**a**^	**692** **±** **81**^**a**^
	VetK	**591** **±** **169**	**647** **±** **147**	**712** **±** **61**	**745** **±** **66**	**746** **±** **65**

Max. angular velocity time (ms)	YgK		**565** **±** **261**^**a**^	**497** **±** **70**^**a**^	520 ± 188	
	VetK		**632** **±** **237**	**540** **±** **62**	536 ± 158	

Max. angular acceleration time (ms)	YgK		**664** **±** **90**^**a**^	**465** **±** **108**^**a**^	551 ± 128	
	VetK		**713** **±** **70**	**496** **±** **78**	577 ± 136	

Time to contact max. angular velocity (ms)	YgK		133 ± 256	201 ± 34	**178** **±** **167**^**a**^	
	VetK		114 ± 213	206 ± 16	**210** **±** **140**	

Time to contact max. linear velocity (ms)	YgK	188 ± 95	165 ± 84	165 ± 34	**60** **±** **33**^**a**^	81 ± 37
	VetK	207 ± 90	178 ± 67	168 ± 14	**60** **±** **5**	78 ± 7

Time to contact max. linear acceleration (ms)	YgK	195 ± 108	142 ± 118	51 ± 62	3 ± 23	6 ± 34
	VetK	155 ± 136	100 ± 111	34 ± 21	1 ± 7	0 ± 0

Time to contact max. angular acceleration (ms)	YgK		34 ± 39	233 ± 81	147 ± 96	
	VetK		33 ± 34	250 ± 54	169 ± 111	

Note: VetK = veteran karate practitioners; YgK = young karate practitioners. Values are mean ± sd. ^**a**^Statistically significant differences between groups (*α* ≤ 0.05).

## Data Availability

The data used to support the findings of this study are provided by tables.
